# Deep brain stimulation may be a viable option for resistant to treatment aggression in children with intellectual disability

**DOI:** 10.1111/cns.14156

**Published:** 2023-03-08

**Authors:** Juan Carlos Benedetti‐Isaac, Loida Camargo, Martin Torres Zambrano, Esther Perea‐Castro, Edgard Castillo‐Tamara, Nicole Caldichoury, Jorge Herrera‐Pino, Yuliana Flórez, María Porto, Norman López

**Affiliations:** ^1^ Medihelp Clinic Cartagena Colombia; ^2^ Facultad de Medicina, Universidad de Cartagena Cartagena Colombia; ^3^ Escuela de Medicina, Universidad del Sinú Cartagena Colombia; ^4^ Universidad de Los Lagos Osorno Chile; ^5^ Florida International University Miami USA; ^6^ Universidad de La Costa Barranquilla Colombia; ^7^ Department of Cognition, Development and Educational Psychology University of Barcelona Barcelona Spain

**Keywords:** aggressive behavior, deep brain stimulation, hypothalamic nuclei, severe intellectual disability

## Abstract

**Introduction:**

Deep brain stimulation (DBS) is a surgical technique used to manage aggression in patients who do not improve despite the use of appropriate drug treatment.

**Objective:**

The objective of this study is to assess the impact of DBS on aggressive behavior refractory to the pharmacological and behavioral treatment of patients with Intellectual Disabilities (ID).

**Methods:**

A follow‐up was conducted on a cohort of 12 patients with severe ID, undergoing DBS in posteromedial hypothalamic nuclei; evaluated with the Overt Aggression Scale (OAS), before the intervention, at 6, 12, and 18 months of medical follow‐up.

**Results:**

After the surgical procedure, there was a significant reduction in the aggressiveness of patients in the follow‐up medical evaluation at 6 months (*t* = 10.14; *p* < 0.01), 12 months (*t* = 14.06; *p* < 0.01), and 18 months (*t* = 15.34; *p* < 0.01), respect to the initial measurement; with a very large effect size (6 months: *d* = 2.71; 12 months: *d* = 3.75; 18 months: *d* = 4.10). From 12 months onward, emotional control stabilized and is sustained at 18 months (*t* = 1.24; *p* > 0.05).

**Conclusion:**

DBS in posteromedial hypothalamic nuclei may be an effective treatment for the management of aggression in patients with ID refractory to pharmacological treatment.

## Graphical abstract

We find a significant decrease in the aggressiveness and functional improvement in the patients treated with DBS.

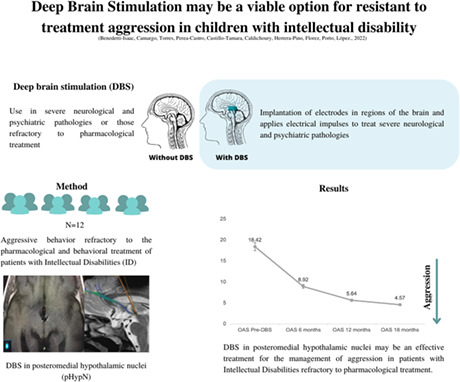

## 1 INTRODUCTION

Aggression is a primitive social behavior essential for defense, protection, and obtaining resources. Studies in humans and other mammals indicate that the amygdala is a key component of a larger neural circuit, including the hypothalamus, that modulates aggressive behavior.^1^, ^2^ It is believed that impulsive forms of aggressive behavior occur when there is hyperactivation of the limbic system, with insufficient control of the prefrontal cortex, generating an overexpression of aggression that results in a major health problem.^3^

Self‐injurious and aggressive behavior can have a significant impact on the quality of life of affected individuals, making it a challenge to care for them.^4^ Severe forms of aggression put the patients´ integrity at risk, along with that of their family members and caregivers. As a result, they are sometimes withdrawn from schools and psychological care programs, due to the refractoriness of the symptoms.^5^ Unfortunately, when aggression is intractable because symptoms are resistant even to pharmacological treatment, patients are subjected to conditions of direct mechanical coercive restraint, such as the use of belts, gloves, and protective helmets, to minimize self‐mutilation, self‐injury, facial disfigurement, and cranioencephalic contusions.^4^, ^6^, ^7^, ^8^ However, intractable aggression is not a clinical condition defined in the diagnostic and statistical manuals of mental disorders. It is a symptom frequently observed in various psychiatric and neurological disorders; it has a high prevalence in patients with neurodevelopmental disorders, such as Intellectual Disability (ID). This is characterized by impairment in cognitive abilities, commonly defined by an IQ <70, and severe deficits in the ability to adapt to the environment and social milieu.^9^ It is estimated that in 45% of patients with ID there is aggressive behavior^10^, ^11^; putting patient safety at risk^2^ and generating discomfort in family members and caregivers.^12^

The main treatment to reduce aggression in ID is pharmacological prescription and behavioral therapy that generates variable results.^13^ However, despite the diversity of drugs and doses used to treat aggression, some patients do not respond adequately to traditional treatment, due to the severity of clinical symptoms and associated brain dysfunction,^14^ responsible for their aggressive behavior. For this limited population of patients with intractable aggression, surgical interventions in the amygdala and hypothalamus have been proposed.^15^

Deep brain stimulation (DBS) is a new and promising method for the treatment of a wide spectrum of clinical conditions^16^, ^17^, ^18^, ^19^, ^20^, ^21^; including patients with uncontrollable aggression.^14^, ^15^, ^22^, ^23^ It consists of the implantation of electrodes in certain regions of the brain, where a neurostimulator applies electrical impulses to treat severe neurological and psychiatric pathologies or those refractory to pharmacological treatment. There are several studies on the role of DBS in aggressive behavior, which have targeted the posterior hypothalamic region. However, clinical data on this treatment modality are still lacking^24^; especially, in pediatric populations where evidence is advancing slowly.^25^ Therefore, we conducted a follow‐up study in 12 adolescent patients with severe ID and intractable aggressive behavior; where a significant reduction (clinical and statistical) of aggressive behavior is demonstrated at 6, 12, and 18 months, following the implementation of DBS.

Aim: To analyze the effectiveness of DBS at the level of posteromedial hypothalamic nuclei (pHypN), in the aggressive behavior of a group of patients with ID, refractory to pharmacological and behavioral treatment for 18 months.

**Hypotheses:** DBS is an adequate and effective neurosurgical technique to reduce intractable aggressive behavior in subjects with severe ID; improving the adaptive functioning and quality of life of these patients.

## 2 METHODS

A follow‐up study of a cohort of 12 patients with severe ID, with symptoms of uncontrollable, impulsive aggression, refractory to psychopharmacological, and behavioral treatment. The children were chosen by the medical board and clinical consensus for the DBS surgical procedure. All participants were evaluated, before the intervention and in clinical follow‐ups at 6, 12, and 18 months, by expert professionals, which included clinical and psychometric assessments of aggressiveness.

### 2.1 Participants

Twelve pediatric patients underwent stereotactic DBS implantation in pHypN bilateral, with the aid of intraoperative cerebral micro‐recording (Icmr). Patients who met the inclusion criteria (Table 1), were operated on by an expert neurosurgeon (JCBI), in a clinical center in the cities of Cartagena de Indias (Medihelp Clinic) and Barranquilla (Clinica Porto Azul), Colombia.

**TABLE 1:** Patient inclusion criteria.

1	Aggressive behavior disorder produces a great deterioration in the patient's activity of daily living
2	High scores on the Overt Aggressiveness Scale (OAS). The scale evaluates four types of aggressive behaviors: verbal aggression, aggression against oneself, aggression against objects, and aggression against other people. The minimum score is 4 and the maximum is 20
3	Score <40 on the global functioning scale
4	Failures in psychopharmacological treatment include at least 2 antipsychotics, a mood‐stabilizing drug, and a benzodiazepine, for at least 3 years
5	Failure of behavioral treatment
6	Medical management of other medical, neurological, and psychiatric disorders
7	Informed consent of the patients or legal guardians
8	Concept of refractoriness of the psychopharmacological and behavioral treatment, at least by two physicians specialized in psychiatry or a medical board of this specialty
9	The medical board of the interdisciplinary group approves the procedure (Neurosurgery, Psychiatry, Neurology, and Neuropsychology).
10	Approval of the institutional ethics committee

### 2.2 Pre and postoperative evaluation

Initially, patients were evaluated by neuropsychology, which included the Wechsler Intelligence for children Scale‐Fourth Edition (WISC‐IV), together with a neurological assessment, to confirm ID and degree of severity. In addition, psychiatric assessment and interview with relatives or legal guardians were included. The patients had a clinical history of unusually increased aggressiveness, at least 5 years of uncontrolled aggression towards others, self‐injury, and refractoriness to pharmacological and psychological treatment.

After the medical evaluations were performed, it was proposed to the relatives to submit the patients to DBS, performed by the neurosurgeon of the clinical center and the medical team. After explaining the implications of the surgery and obtaining informed consent from parents and legal guardians, the patients were examined before surgery by psychiatry and neuropsychology, where the Overt Aggression Scale (OAS) [26] was administered in a non‐blinded manner. The OAS^26^ has four types of aggressive behaviors: verbal aggression, aggression against self, aggression against objects, and aggression against other people. The specific type of each aggression is tested in each category, ranging from mildly threatening forms of aggression, for example, kicking, yelling, or slamming a door, to more severe forms, resulting in injury or loss of consciousness. A weighted score is assigned to each category, according to the original OAS design. The weighted score for each of the four categories is summed to obtain the instrument's aggression score (e.g., 3 + 3 + 4 + 5 = 15). The minimum score is 4 (low‐level) and the maximum score of the instrument is 20 (the highest score of aggressiveness). Subsequently, bilateral electrodes were implanted in pHypN with the help of intraoperative brain micro registration. Medical check‐ups were performed at 6, 12, and 18 months, during which the OAS was re‐administered. In addition, parents or guardians were interviewed about the aggressive behavior of the patients before and after DBS.

### 2.3 Surgical technique

**FIGURE 1:**
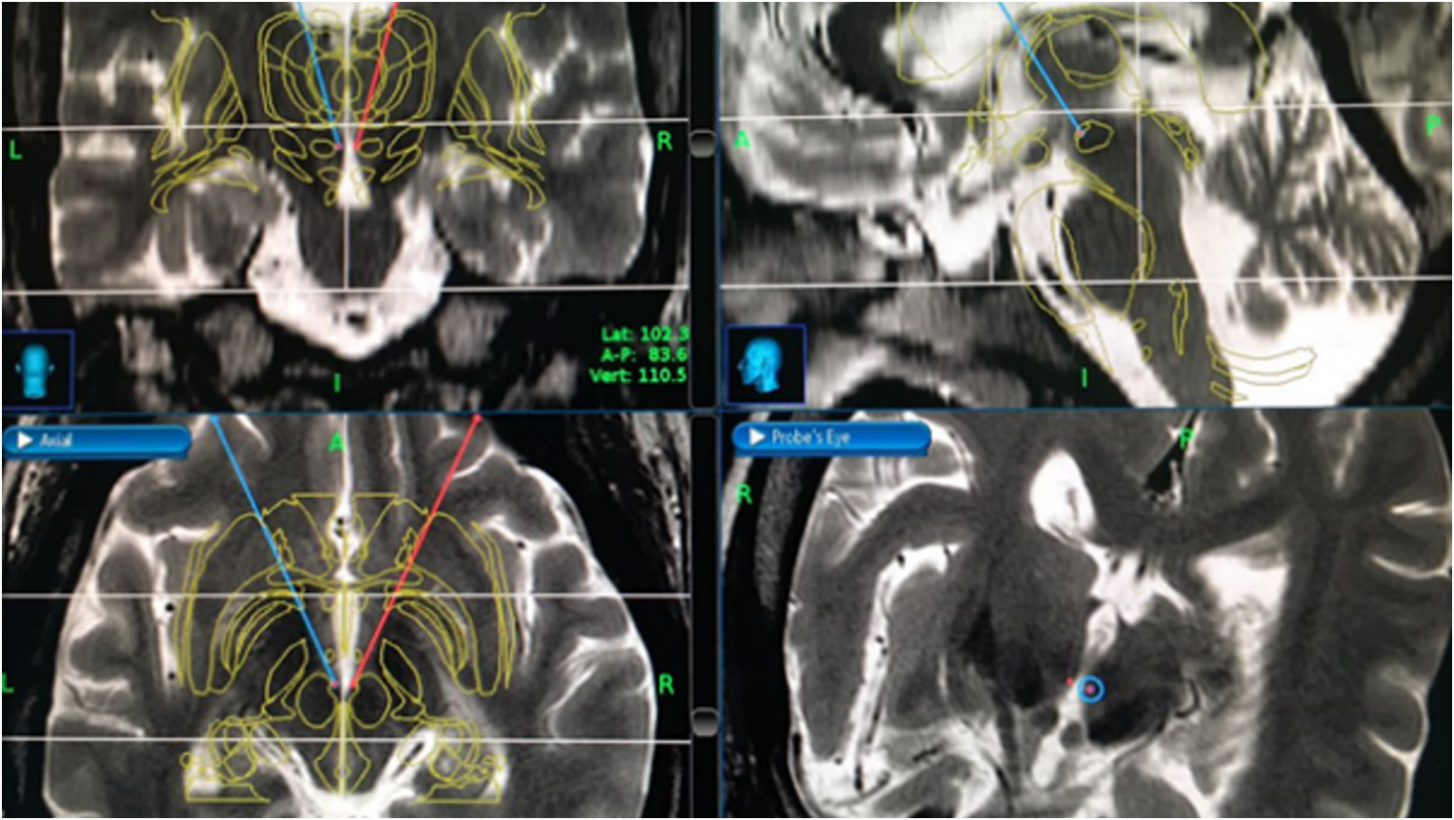
Stereotactic planning.^36^

Stereotactic implantation was performed with the Leksell frame (Elekta Inc), under general anesthesia with intravenous dexmedetomidine, and local nerve block. Contrasted stereotactic volumetric brain computed tomography images were acquired at a thickness of 1 mm; and fused with preoperative volumetric brain magnetic resonance imaging with gadolinium, thin‐slice 1 mm. Surgical implantation was performed with the aid of Stealth Station Cranial Software, Medtronic, USA; and Inomed software, Emmendingen, Germany (Figure 1). We used the technique of ^22^, ^23^, ^27^ for the stereotactic coordinates: 2 mm lateral, 3 mm posterior, and 5 mm inferior to the mid‐commissural point (MCP) of the anterior commissure–posterior commissure (AC–PC) intercommissural line distance, and correlation with functional stereotactic atlas (Schaltenbrand and Warren human brain atlas).

**FIGURE 2:**
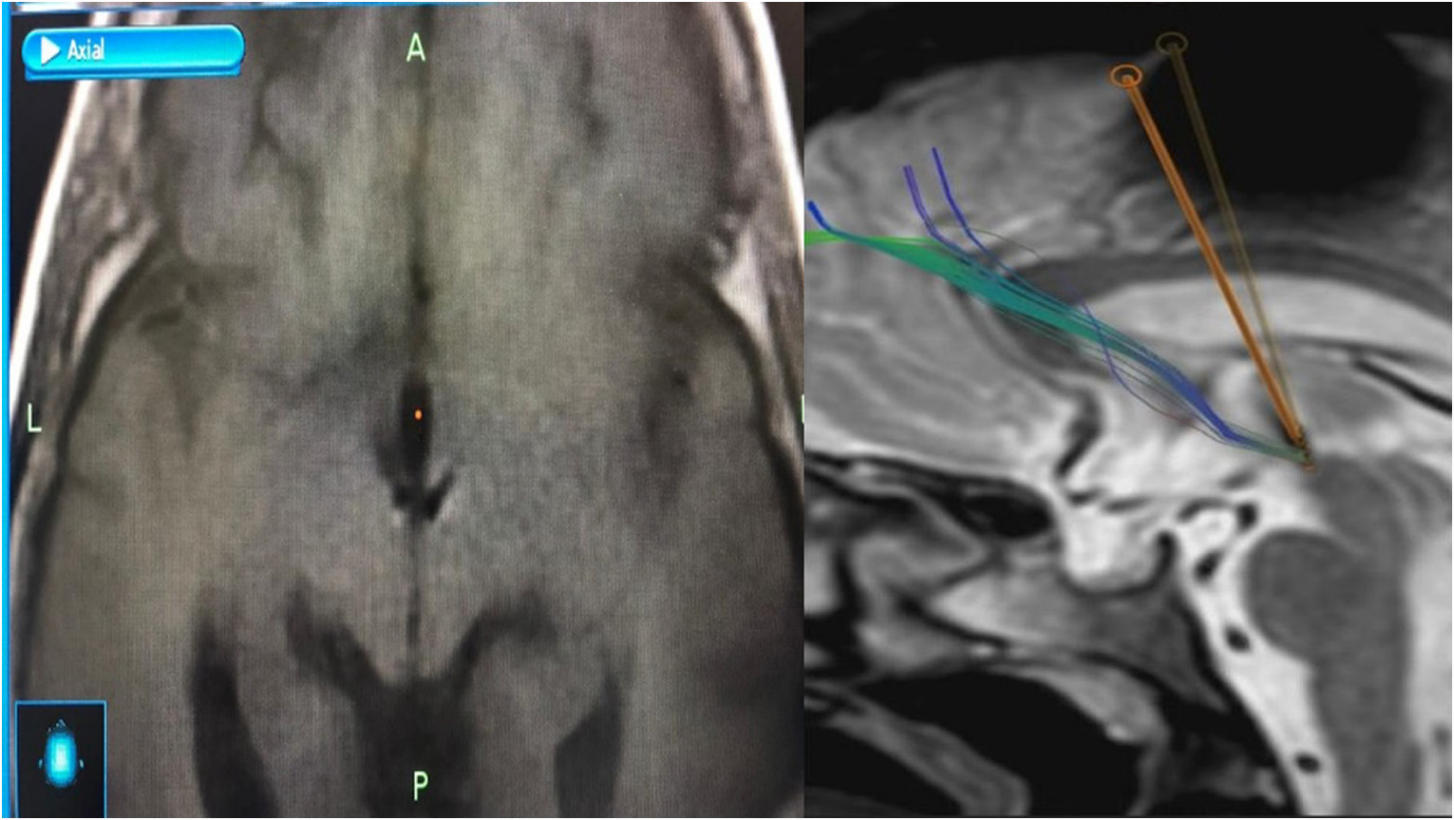
Tw1 MRI/CT‐S intraOp fusion showing deep brain electrodes implanted in the pHypNs.

After anatomical‐functional localization of the target point, bilateral deep brain microelectrodes were implanted stereotactically, with neurophysiological monitoring and intraoperative stimulation tests. Then, definitive deep brain electrodes (3389, Medtronic, MA, U.S.A) were implanted at the level of the pHypN, with the placement of a neurostimulation generator (Medtronic, MA, USA) (Figure 2). A default trajectory was established through the brain at 60° from the AC–PC line in the sagittal projection and 10° from vertical in the coronal projection. This trajectory was visualized in the volumetric magnetic resonance imaging (MRI) study using navigation views.

After defining the target point, the final DBS electrode was implanted (3389, Medtronic, Minneapolis, MN, USA) was implanted, with a previously biplanar radiographic marking of the target point. The DBS electrodes were implanted bilaterally in the pHypN, using the same stereotactic technique. After 1 month of electrode implantation, stimulation with 2 V, 90 μs, pulse width, and 180 pulses/s were started. The surgery was performed satisfactorily, complying with biosafety measures to prevent Covid‐19 infections, as well as bleeding associated with the procedure. No postoperative complications were observed, and the patients were discharged on the second day of hospitalization.

### 2.4 Statistical analysis

First, descriptive analyses of skewness and kurtosis were performed, and the Kolmogorov–Smirnov tests were used to estimate the statistical normality of the data. Secondly, to analyze the mean performances in the aggressiveness scale, between the first clinical control before DBS and the 3rd medical follow‐ups, repeated measures analysis of variance (ANOVA) was used, fulfilling the assumptions of homogeneity of variances of Mauchly's test of sphericity and a post hoc analysis with Bonferroni correction. Cohen's omega‐square and *d* were also used as a measure of effect size in those results that were significant, based on the proposal of ^28^. Their interpretation is a small effect (0.15–0.40), a medium effect (0.40–0.75), and a large effect (+ 0.75). All analyses were processed with the SPSS 25. software.

### 2.5 Ethics statement

All participants, family members, or their legal guardian's signed informed consents for the clinical procedure and the study. Medical meetings were held to select the candidates for the surgical procedure. Clinical controls were performed by a multidisciplinary team. National and international ethical standards were complied with following the Helsinki Declaration of 1975 revised in 2008.

## 3 RESULTS

**FIGURE 3:**
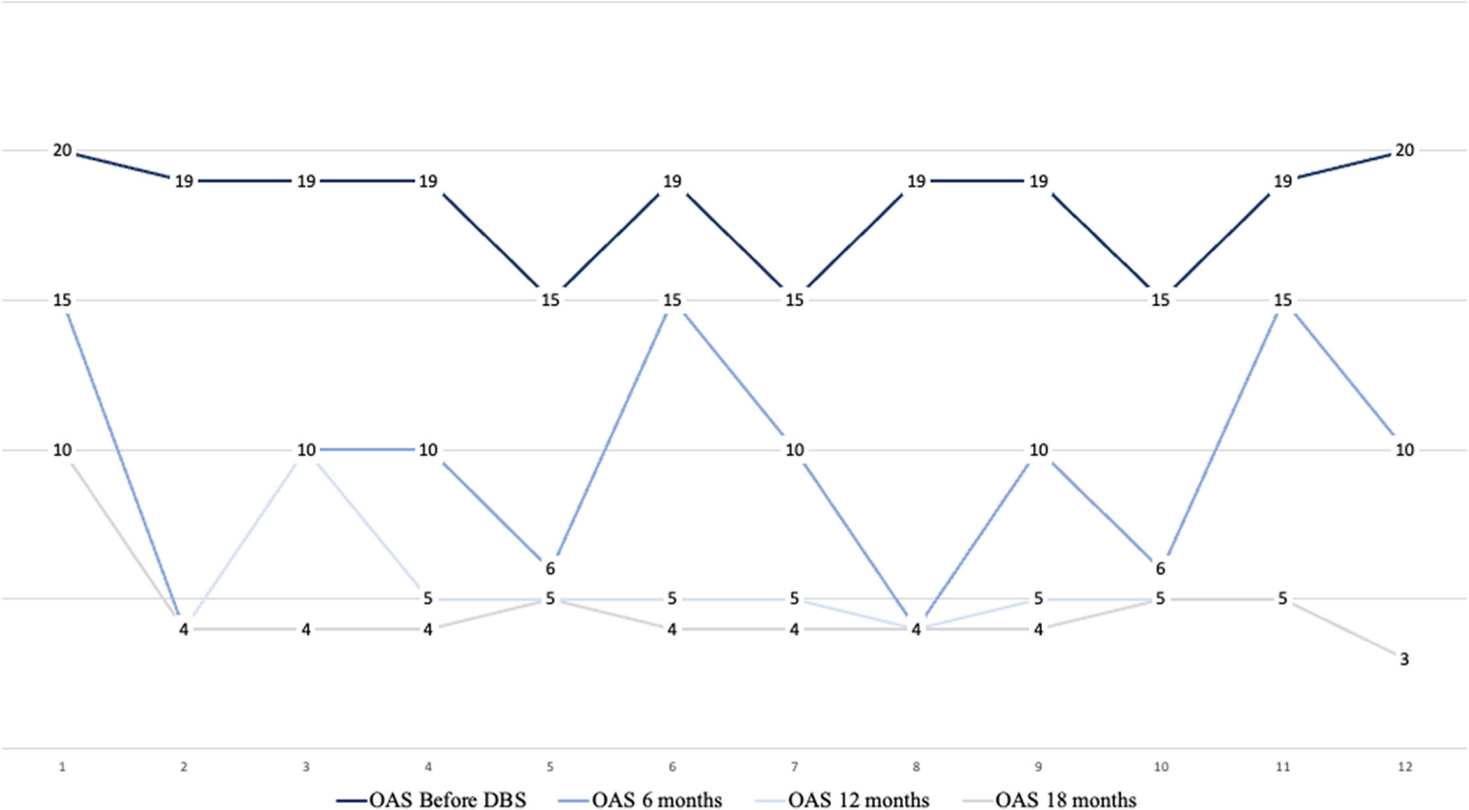
Changes in aggressiveness (OAS) of patients pre‐ and post‐DBS.

The average age of the participants was 15.34 years (SD = 1.80). In the neuroimaging study, the finding of cortico‐subcortical atrophy was widespread. None of the patients experienced any surgical complications. Regarding the results of the aggressiveness assessment, before DBS, high mean performances were found in the OAS (ME = 18.42; SD = 3.03). However, at post‐surgical follow‐up at 6 (ME = 8.92; SD = 3.58), 12 (*M* = 5.64; SD = 2.00), and 18 months (ME = 4.57; SD = 0.93), all patients undergoing DBS achieved a significant decrease in aggressiveness (Figure 3). ANOVA confirmed significant changes in each of the clinical follow‐ups (*p* < 0.01), with a high omega squared (*ω* ^2^ = 0.876), in favor of the surgical technique. Table 2 shows the intragroup post hoc. This reflects significant intra‐group differences in the mean aggressiveness before DBS, with large effect sizes; except for the 12‐ and 18‐month follow‐up, where no significant differences were found. After DBS, in the first follow‐up visit, a significant reduction in aggressiveness is evidenced, which continues at 12 months. However, after 18 months, the curve of aggressiveness decreased and flattened. It seems that after the 18th month, emotional control is maintained, benefiting the quality of life and social inclusion of the patients.

**TABLE 2:** Intra‐group comparisons OAS pre‐ and post‐BDS with post hoc.

		Mean difference	Se	*t*	*Cohen's d*	*p* bonf
Before DBS	OAS 6 m	8.500	0.838	10.145	2.711	<0.001
	OAS 12 m	11.786	0.838	14.067	3.759	<0.001
	OAS 18 m	12.857	0.838	15.345	4.101	<0.001
OAS 6 m	OAS 12 m	3.286	0.838	3.922	1.048	0.002
	OAS 18 m	4.357	0.838	5.200	1.390	<0.001
OAS 12 m	OAS 18 m	1.071	0.838	1.279	0.342	1.000

Abbreviations: P, *p*‐Value; Se, Sensitivity; *t*, Student's test.

## 4 CONCLUSION

We conducted a clinical follow‐up study for 18 months in 12 patients with symptoms of severe impulsive, uncontrolled aggression associated with a neurodevelopmental disorder, who underwent DBS because they did not respond adequately to pharmacological and psychological treatment. The selected patients were evaluated by psychiatry and neuropsychology, before DBS and in 3 subsequent controls (at 6, 12, and 18 months). In each clinical assessment was analyzed the aggressiveness of the patients using the OAS as an objective contrast measure.

Preliminary results revealed a significant reduction in the aggressiveness of the patients after having undergone DBS. In each of the medical controls (6, 12, and 18 months), a substantial decrease in aggressive behavior was observed. In contrast with the high indicators of aggressiveness found in the first medical control, performed before the application of the neurosurgical technique. Most interesting was that between the clinical control performed at 12 and 18 months, concerning the medical control at 6 months, significant differences were identified in the mean scores on the aggressiveness scale. In both cases, with very‐large effect sizes. Revealing a sustained and accelerated decrease in aggressiveness and suggesting that DBS was responsible for the decrease in aggressiveness and functional improvement in the patients.

Globally, the efficacy of DBS of the posterior hypothalamus for aggressive behavior is advancing steadily. Sano et al.^29^ and^30^ reported good results with DBS in bilateral lesions at the level of the posteromedial hypothalamic nucleus in patients with pathological aggression. Franzini et al.^22^, ^27^ described in two major publications, the treatment of patients to whom DBS was applied, at the level of the posteromedial hypothalamic nuclei, achieving good control of aggressive behavior.

Rizzi described the benefits of DBS for aggressive behavior in one case.^10^ Our group analyzed the impact of DBS in pHypN on seizure frequency in nine patients with drug‐resistant epilepsy and pharmacologically intractable aggressive behavioral symptoms, reporting significant improvement in both cases.^31^ Recently, a follow‐up study of seven patients with pathological aggressiveness reported benefits in five patients who received DBS in the posteromedial hypothalamus. Brain areas involved in the stimulation field were identified; by stimulating the centromedial nucleus, prefrontal thalamocortical circuits were activated, reducing aggressiveness in a small group of patients.^32^

Unfortunately, the evidence in favor of DBS for intractable aggressive behavior in children and adolescents with neurodevelopmental disorders, such as ID, is scarce.^25^ Hence, the need for larger follow‐up studies and prospective analyses, to define pHypN as a target in different contexts; incorporating rigorous selection and follow‐up criteria, carefully designed for children and adolescents. In this context, before the Covid‐19 pandemic, our working group,^33^ performed the largest follow‐up study reported in the literature; analyzing the impact of DBS in the hypothalamic posteromedial nuclei in 19 pediatric patients with ID and intractable aggression; demonstrating a significant reduction of aggressive behavior in 18 months of follow‐up, with a clinical and statistical magnitude, explained by the surgical technique. The results of the study point in the direction that DBS in pHypN modulates the electrical activity of afferents in the frontal lobe (orbitofrontal cortex and anterior cingulate gyrus) and temporal lobe (amygdala). This improved the efficiency of processing in the orbital frontal/cingulate cortex, attenuating the hyperactivity of the amygdala; restoring serotonergic activity, with the consequent reduction of aggressive behavior; which would explain the results of this series of cases.^10^, ^22^

**FIGURE 4:**
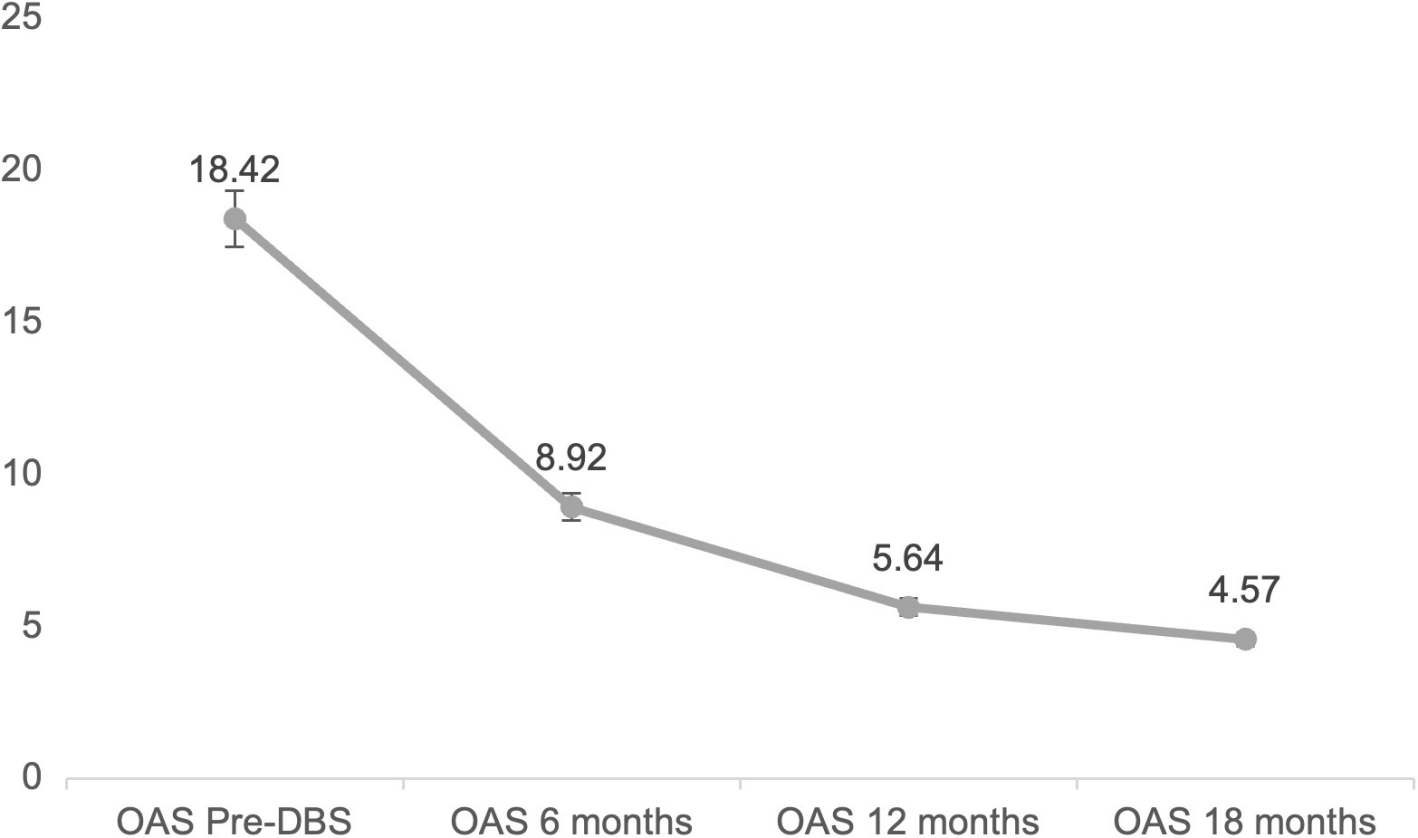
Variations in patient aggressiveness after DBS.

Therefore, in this opportunity, we present the results of a second follow‐up study, conducted during the Covid‐19 pandemic (2021), in a pediatric population with neurodevelopmental disorder and intractable aggression. Unlike the first study,^33^ we incorporated more objective measures of intelligence, graded the level of ID and adaptive functioning, and more accurately estimated the degree of aggressiveness of the patients; this to have a clean sample of subjects in severe stages of ID. Likewise, details of the clinical procedure and surgical technique were refined. To minimize some effects described in the literature of the operative site^34^ and behavioral regression.^35^ As a result, we found a clear reduction in aggressive behavior sustained over time. In each clinical control in which the subjects were assessed (6, 12, and 18 months), the objective indicators of aggressiveness decreased significantly. In addition, the report of the relatives or guardians confirmed the findings. Physical aggression and self‐injury were significantly reduced in all patients. After 18 months of follow‐up, we observed that the accelerated decrease in the aggressiveness curve (see Figure 4), objectified by the OAS and confirmed by family members, began to flatten out and become sustained; thus, we consider that patients and their families experience higher levels of functionality and quality of life.

However, considering that deep brain stimulation is a surgical procedure, we must recognize that there are risks and limitations. Franzini et al.^27^ described one patient who died from intraparenchymal and intraventricular hemorrhage, 3 days after the intervention. Analyzed the risk factors and complications of DBS,^34^ using a supervised learning algorithm with a clinical accuracy of 0.86, to predict any complications associated with DBS, such as infection (AUC = 0.97), return to the operating room (AUC = 0.88), and complication during the following 12 months (AUC = 0.91). They showed that the risk of complication was related to the institution where the surgery was performed (odds ratio [OR] = 0.44, confidence interval [CI] = 0.25–0.78). Patients with diabetes were almost 3 times more likely to return to the operating room (OR = 2.78, CI = 1.31–5.88), and patients with a history of smoking were 4 times more likely to experience postoperative infection (OR = 4.20, CI = 1.21–14.61).^34^ Recently, Tsuboi et al.^35^ reported a suboptimal response of 8% to deep brain stimulation in patients with early‐onset dystonia carrying DYT1 mutation in a multi‐country cohort. They identified some phenotypic characteristics of clinical conditions, such as the age of onset Table 1, disease progression, severity level, and cranial involvement which explained the secondary worsening in these patients after DBS. For this reason, rigorous protocols for the selection and inclusion of patients in the surgical procedure and more frequent post‐surgical follow‐ups should be considered, making the post‐DBS results of pHyp safer.

In some studies, deep brain stimulation of pHypN has been used in aggressive behaviors refractory to pharmacological and behavioral treatment. In our follow‐up study, a clear reduction of aggressive behavior was achieved, remaining constant until 18 months of follow‐up in 12 patients with severe ID. These results suggest that deep DBS is a successful treatment option for patients with uncontrolled aggression resistant to traditional treatments.

## FUNDING INFORMATION

Universidad de la Costa and Medihelp Clinic.

## CONFLICT OF INTEREST STATEMENT

No conflicts of interest have been declared.

## Data Availability

We confirm the absence of shared data.
